# A negative feedback loop between fibroadipogenic progenitors and muscle fibres involving endothelin promotes human muscle fibrosis

**DOI:** 10.1002/jcsm.12974

**Published:** 2022-03-22

**Authors:** Mona Bensalah, Laura Muraine, Alexis Boulinguiez, Lorenzo Giordani, Victorine Albert, Victor Ythier, Jamila Dhiab, Alison Oliver, Valentine Hanique, Teresa Gidaro, Sophie Perié, Jean Lacau St‐Guily, Aurélien Corneau, Gillian Butler‐Browne, Anne Bigot, Vincent Mouly, Elisa Negroni, Capucine Trollet

**Affiliations:** ^1^ Sorbonne Université Inserm, Institut de Myologie, Centre de Recherche en Myologie Paris France; ^2^ Department of Otolaryngology‐Head and Neck Surgery Tenon Hospital, Assistance Publique des Hôpitaux de Paris, Faculty Medicine Sorbonne University Paris France; ^3^ Department of Otolaryngology Head and Neck Surgery Com Maillot‐Hartmann Clinic Neuilly Sur Seine France; ^4^ Department of Otolaryngology‐Head and Neck Surgery Rothschild Foundation Hospital and Sorbonne University Paris France; ^5^ UMS037, PASS, Plateforme de Cytométrie de la Pitié‐Salpêtrière CyPS Sorbonne Université Paris France

**Keywords:** Human, FAPs, Skeletal muscle, Pharyngeal muscle, Fibrosis, ECM, Regeneration, Endothelin, TGFβ

## Abstract

**Background:**

Fibrosis is defined as an excessive accumulation of extracellular matrix (ECM) components. Many organs are subjected to fibrosis including the lung, liver, heart, skin, kidney, and muscle. Muscle fibrosis occurs in response to trauma, aging, or dystrophies and impairs muscle function. Fibrosis represents a hurdle for the treatment of human muscular dystrophies. While data on the mechanisms of fibrosis have mostly been investigated in mice, dystrophic mouse models often do not recapitulate fibrosis as observed in human patients. Consequently, the cellular and molecular mechanisms that lead to fibrosis in human muscle still need to be identified.

**Methods:**

Combining mass cytometry, transcriptome profiling, *in vitro* co‐culture experiments, and *in vivo* transplantation in immunodeficient mice, we investigated the role and nature of nonmyogenic cells (fibroadipogenic progenitors, FAPs) from human fibrotic muscles of healthy individuals (FibM^CT^) and individuals with oculopharyngeal muscular dystrophy (OPMD; FibM^OP^), as compared with nonmyogenic cells from human nonfibrotic muscle (M^CT^).

**Results:**

We found that the proliferation rate of FAPs from fibrotic muscle is 3–4 times higher than those of FAPs from nonfibrotic muscle (population doubling per day: M^CT^ 0.2 ± 0.1, FibM^CT^ 0.7 ± 0.1, and FibM^OP^ 0.8 ± 0.3). When cocultured with muscle cells, FAPs from fibrotic muscle impair the fusion index unlike M^CT^ FAPs (myoblasts alone 57.3 ± 11.1%, coculture with M^CT^ 43.1 ± 8.9%, with FibM^CT^ 31.7 ± 8.2%, and with FibM^OP^ 36.06 ± 10.29%). We also observed an increased proliferation of FAPs from fibrotic muscles in these co‐cultures in differentiation conditions (FibM^CT^ +17.4%, *P <* 0.01 and FibM^OP^ +15.1%, *P <* 0.01). This effect is likely linked to the increased activation of the canonical TGFβ‐SMAD pathway in FAPs from fibrotic muscles evidenced by pSMAD3 immunostaining (*P* < 0.05). In addition to the profibrogenic TGFβ pathway, we identified endothelin as a new actor implicated in the altered cross‐talk between muscle cells and fibrotic FAPs, confirmed by an improvement of the fusion index in the presence of bosentan, an endothelin receptor antagonist (from 33.8 ± 10.9% to 52.9 ± 10.1%, *P* < 0.05).

**Conclusions:**

Our data demonstrate the key role of FAPs and their cross‐talk with muscle cells through a paracrine signalling pathway in fibrosis of human skeletal muscle and identify endothelin as a new druggable target to counteract human muscle fibrosis.

## Introduction

Fibrosis is defined as the excessive accumulation of extracellular matrix (ECM) components (particularly collagen) as a result of a failed tissue‐repair process and can occur in many organs, including the lung, liver, heart, skin, kidney, and muscle. In skeletal muscle, fibrosis occurs in response to trauma, aging, or muscular dystrophies and hinders the muscle regeneration process and the efficiency of therapeutic strategies.^1^

In mouse skeletal muscle, fibrosis involves humoral factors, such as TGFβ, a key driver of ECM remodelling,^2^ pro‐inflammatory and anti‐inflammatory cytokines, and growth factors such as CTGF and PDGF.^1^ At the cellular level, fibrosis involves inflammatory cells, fibro/adipogenic progenitors (FAPs, the major collagen‐producing cell type within the stromal tissue microenvironment^3^, ^4^), and muscle stem cells, that is, satellite cells.^5^ While muscle fibrosis in mice has been clearly defined, very little is known about human muscle fibrosis and the molecular and cellular factors triggering it.^6^, ^7^ Mice are not men, and although mice do provide models to get insight into different biological processes, there are substantial differences, particularly in the case of fibrosis. For example, if we compare the muscle of patients with Duchenne muscular dystrophy (DMD) to the muscle of the mdx mouse model of DMD, there is very little fibrosis in the mdx,^8^ whereas in human patients, the whole of the muscle becomes progressively replaced by fibrotic tissue over the time course of the disease.^6^ This is also the case for oculopharyngeal muscular dystrophy (OPMD): there is very little fibrosis in mouse models compared with human patients.^9^, ^10^ Because several cell types are involved, many aspects of the cellular cross‐talk that contributes to the initiation and maintenance of fibrosis in human skeletal muscle need to be elucidated in order to identify targets to counteract this process. In particular, although FAPs in mouse muscle have been characterized during regeneration,^11^ disease,^12^ and during aging,^13^ little is known about the role of these cells in human muscle fibrosis.^14^ In fibrotic muscle from dystrophic mice, TGFβ has been found to drive the differentiation of FAPs into matrix‐producing cells.^15^ In mice, mesenchymal cells (FAPs,^3^, ^4^ TCF7L2 + fibroblasts,^16^ and pericytes^17^) have been shown to support muscle repair or participate in fibrosis,^3^, ^4^, ^11^, ^12^, ^18^, ^19^ but whether these cells are involved in the human fibrosis and which pathways are active had not been investigated in detail.^14^

We have previously shown that the cricopharyngeal muscle (CPM) in healthy aged controls is characterized by a high level of ECM deposition (20% of the muscle) compared with limb muscle.^9^ This ECM deposition becomes excessive (40% of the muscle) in the CPM of patients suffering from OPMD, a slow‐progressing, late‐onset degenerative muscle disorder mainly characterized by dysphagia and ptosis.^9^ Interestingly, in addition to OPMD, the progressive loss of pharyngeal muscle function is frequently encountered in other myopathies (inclusion body myositis, mitochondrial myopathies …) as well as in aged subjects, a condition called achalasia.^20^ In this study, we investigated the cellular actors of both physiological (high level of ECM in control CPM) and pathological (excessive ECM deposition in OPMD CPM) human muscle fibrosis by comparing the CPM from control subjects and patients with OPMD to nonfibrotic muscles from control subjects. First, we isolated and characterized the CD56− cell fraction among the nonmyogenic cells in muscles from these three sources. We show that in culture, these CD56− nonmyogenic cells exhibit the characteristics defining FAPs. Then, we demonstrate that human FAPs isolated from both healthy and pathological fibrotic skeletal muscles present characteristics distinct from those observed in cells isolated from healthy nonfibrotic control muscles. In particular, FAPs isolated from fibrotic muscle show a strikingly higher proliferative capacity and can inhibit muscle differentiation via ECM protein production. Furthermore, in addition to the known profibrogenic cytokine TGF‐β, we identified endothelin as a key profibrotic player in this process in human muscle. Altogether, our data demonstrate the key role of FAPs and their cross‐talk with muscle cells through a paracrine signalling pathway in fibrosis in human muscle and identify endothelin as a target for future potential antifibrotic interventions.

## Methods

### Cell culture

Human muscle biopsies (control nonfibrotic muscle: M^CT^, control non‐dystrophic fibrotic muscle: FibM^CT^, and OPMD fibrotic muscle: FibM^OP^) used in this study (*Table* 1) were obtained via Myobank‐AFM, affiliated with EuroBioBank, in accordance with European recommendations and French legislation (authorization AC‐2019‐3502). Cells were isolated by explant culture method: muscle biopsies were minced, and explants were plated onto dishes coated with fetal bovine serum (FBS) (Invitrogen), as previously described.^21^ Isolated cells were resuspended in FBS containing 10% DMSO and transferred into cryogenic storage vials, frozen at −80°C using a freezing container. Cell thawing was performed quickly using pre‐warmed growth medium. Next day, medium was removed to get rid of the DMSO, and cells were trypsinized, counted, and replated at 37°C in growth medium consisting of 199 medium (Life Technologies) and Dulbecco's modified Eagle's medium (DMEM, Life Technologies) at a 1:4 ratio supplemented with 20% FBS, 25 μg/mL fetuin (Life Technologies), 0.5 ng/mL bFGF (Life Technologies), 5 ng/mL EGF (Life Technologies), 5 μg/mL insulin (Sigma‐Aldrich), and 50 μg/mL gentamycin (Life Technologies) in a humid atmosphere containing 5% CO_2_. The cells were labelled with CD56 antibody‐coupled microbeads (130‐050‐401, Miltenyi Biotec), and CD56+ and CD56− cell fractions were separated using magnetic activated cell sorting (MACS) according to the manufacturer's instructions. The myogenic purity was monitored throughout the whole study and for each experiment by immunocytochemistry after fixation 10 min with pure ethanol using an antibody against desmin (clone D33, Dako), which is exclusively expressed in myogenic cells. At least 500 cells per condition were counted. Cells named ‘CD56− cells’ or ‘nonmyogenic cells’ were always <1.5% desmin‐positive; cells named ‘CD56+ cells’ or ‘myogenic cells’ were always at least 95% desmin‐positive. Uncoated petri dishes (353003, Corning‐Falcon) were used for cell amplifications. The number of divisions was calculated according to the formula log (*N/n*)/log 2, where *N* is the number of cells counted and *n*, the number of cells initially plated. Lifespan and proliferative status of cells used in this study have been carefully monitored to make sure cells do not reach pre‐senescence for any of the conditions. For the assessment of the cells surface area, pictures were taken with a light microscope and analysed using ImageJ software.

**Table 1:** Muscle biopsies used in this study

Nomenclature	Muscle	Number
M^CT^	Deltoid	3
	Paravertebral	2
	Quadriceps	5
	Sternocleidomastoid	17
	Tensor Fascae Latae	3
	Tibialis Anterior	1
FibM^CT^	Cricopharyngeal	19
FibM^OP^	Cricopharyngeal	25

### Cell transplantation

Rag2^−/−^Il2rb^−/−^ immunodeficient mice (2–3 months old) were used as recipients of transplanted human nonmyogenic cells. The mice were anaesthetized by an intraperitoneal injection of 80 mg/kg ketamine hydrochloride and 10 mg/kg xylazine. This study was carried out in strict accordance with the legal regulations in France and according to the ethical guidelines for animal research of the European Union. The protocol was approved and delivered by the French Ministry of Higher Education and Scientific Research (number: 2021072217421004). The TAs (tibialis anterior) of the mice were subjected to three freeze lesion cycles of 10 s in order to trigger regeneration, then 15 μL of 1.4 × 10^5^ FAPs in PBS were injected immediately after cryodamage as previously described^21^ and 4 and 8 days later. The mice were sacrificed by cervical dislocation 1 month after the first injection, and the TAs were collected, snap frozen in isopentane, and stored at −80°C for further analyses.

### Muscle histology

xHuman muscle biopsies and mouse TAs were mounted in tragacanth gum (6% in water; Sigma‐Aldrich) placed on a cork support and snap frozen in liquid nitrogen‐cooled isopentane. Transverse serial cryosections (5 μm thick) made with a cryostat (Leica CM1850) were processed to staining. To assess tissue morphology and visualize fibrotic tissue and connective tissue by light microscopy (Leica DMR microscope equipped with a Nikon DS‐Ri1 camera), the sections were stained with haematoxylin, eosin, and Sirius red. The percentage of fibrosis was assessed from three to seven fields per sample, using ImageJ software, and expressed as a percentage of the total area analysed.

### Coculture experiments

Nonmyogenic and myogenic cells were seeded together at a 30%/70% ratio and a final density of 21 000 cells/cm^2^ in μ‐Slide 8 Well (80826, Ibidi) plates. Once the cells adhered, the growth medium was replaced with DMEM, 10 μM EdU and 50 μg/mL gentamycin. To study the effect of bosentan on the fusion index, 10 μM bosentan (SML1265, Sigma‐Aldrich) was added immediately after growth medium removal and 3 days later. The cells were fixed on day 5 in PBS 4% paraformaldehyde (PFA) for 10 min at room temperature (RT). EdU labelling was performed with the Click‐iT reaction cocktail from the Click‐iT™ EdU Cell Proliferation Kit (C10338, Life Technologies) according to the manufacturer's instructions. The fusion index was calculated as the ratio between the number of nuclei per desmin‐positive myotube (>2 nuclei) and the total number of nuclei. All images were taken on random fields and quantified blinded on at least 500 nuclei per condition.

### Tranforming growth factor β and endothelin‐1 experiments

Cells were plated at 10 000 cells/cm^2^ in μ‐Slide 8 Well plates (80826, Ibidi). For the assessment of the basal level of TGFβ, cells were rinsed and serum‐starved for 2 h in DMEM before fixation in PBS 4% PFA for 10 min. pSmad3 immunostaining was performed with anti‐Smad3 (phospho S423 + S425) antibody (Ab52903, Abcam). For the ET‐1 treatment experiments, cells at 70–80% confluence were rinsed and treated for 3 days with proliferation medium containing 1% FBS and DMSO or 100 ng/mL ET‐1 (E7764, Sigma‐Aldrich) ± 10 μM bosentan (SML1265, Sigma‐Aldrich). To assess proliferation, EdU (10 μM) was added on day 2, and the cells were fixed 24 h later. EdU labelling was detected as described above. To assess ECM secretion, COL7A1 immunofluorescence was performed as described in the immunofluorescence section.

All images were taken on random fields and quantification was performed blinded on at least 500 nuclei per condition.

### RNA extraction and reverse transcription

RNA from frozen muscle sections or cell pellets was extracted using TRIzol reagent (Invitrogen) according to the manufacturer's instructions. The concentration of RNA was determined with a NanoDrop® spectrophotometer ND‐1000, and the quality was assessed with an Agilent 2100 bioanalyzer. RNA was reverse transcribed using M‐MLV (Invitrogen) according to the manufacturer's instructions.

### Transcriptome analysis

Illumina BeadChip Human HT‐12 v4 images were scanned with Bead‐Scan software, generating .idat files, and probe summary profiles were obtained using the illuminaio package. Background correction and normalization were performed using the limma neqc function. Probes were filtered out according to their *P*‐value of detection. Fold change and Benjamini–Hochberg corrected *P*‐value thresholds were set to 1.5 and 0.05.

### Protein secretion prediction

Differentially expressed genes were analysed with the Retrieve/ID mapping tool from the UniProt database to obtain the corresponding reviewed protein sequences. These sequences were then used to obtain information about the presence of signal peptides, transmembrane domains, and unconventional protein secretion using OutCyte v1.0^22^ prediction tools (*Table* S1a,b).

### CyTOF analysis

Nonmyogenic cells were stained with cell‐ID Intercalator‐103Rh (Fluidigm) at 1:2000 for 15 min, then washed in PBS containing DNase (Roche; 1:100), EDTA (Invitrogen; 1/250), resuspended in PBS at a concentration of 3 × 10^6^/50 μL and stained with anti‐CD184 (CXCR4) antibody for 15 min at 37°C. A cocktail of antibodies (*Table* 2) was added to the 3 × 10^6^ cells. After 45 min at RT, cells were washed in PBS containing DNase and EDTA and fixed in PBS 2% PFA for 15 min. The cells were then washed, prechilled on ice for 10 min, and permeabilized in cold methanol for 15 min. The cells were washed twice and stained with LAP antibodies for 45 min at RT. The cells were then washed and incubated overnight at 4°C with intercalator buffer (Intercalator Ir, Fluidigm, 500 mM in 4% PFA). Cells were washed once with PBS, then twice with deionized water and resuspended in deionized water containing standard normalization EQ beads (Fluidigm) at cell concentration adjusted to 5.10^5^ cells/mL. Acquisition was carried out on a CyTOF Helios instrument. The output FCS files were normalized with MATLAB‐based normalization software. Removal of peaks corresponding to debris, dead cells, and doublets was performed on the Cytobank platform (Fluidigm). The data corresponding to residual CD56+ cells (between 0% and 1.3%) were removed, and the analysis was performed with the other 36 markers (*Table* 2). A UMAP plot was obtained from concatenated data from three to four patients for each condition on Cytofkit2.

**Table 2:** Antibody panel used for CyTOF

Antigen	Metal Tag	Clone	Company	Cat#
CD9	171Yb	SN4 C3‐3A2	Fluidigm	3171009B
CD10	156Gd	HI10a	Fluidigm	3156001B
CD13	147Sm	WM15	Fluidigm	3147014B
CD15	172Yb	W6D3	Fluidigm	3172021B
CD24	169Tm	ML5	Fluidigm	3169004B
CD29	167Er	Mar4	BD*	555442
CD31	144Nd	WM59	Fluidigm	3144023B
CD36	152Sm	5‐271	Fluidigm	3152007B
CD41	89Y	HIP8	Fluidigm	3089004B
CD44	166Er	BJ18	Fluidigm	3166001B
CD47	209Bi	CC2C6	Fluidigm	3209004B
CD49a	146Nd	SR84	BD*	559594
CD49b	142Nd	12F1	BD*	555668
CD49c	141Pr	C3 II.1	BD*	556024
CD49d	174Yb	9F10	Fluidigm	3174018B
CD49e	162Dy	IIA1	BD	555615
CD49f	164Dy	GoH3	BD*	3164006B
CD51/61	170Er	23C6	BD*	555504
CD56	176Yb	NCAM16.2	Fluidigm	317600B
CD61	165Ho	VI‐PL2	Fluidigm	3165010B
CD71	148Nd	M‐A712	BD*	555534
CD73	168Er	AD2	Fluidigm	3168015B
CD81	145Nd	5A6	Fluidigm	3145007B
CD82	158Gd	ASL‐24	Fluidigm	3158025B
CD90	161Dy	5E10	Fluidigm	3161009B
CD98	159 Tb	UM7F8	Fluidigm	3159022B
CD104	173Yb	58XB4	Fluidigm	3173008B
CD105	151Eu	166707	R&D systems*	MAB10971
CD106	143Nd	51‐10C9	BD*	555645
CD138	150Nd	DL‐101	Fluidigm	3150012B
PDGFRb	149Sm	28D4	BD*	558820
CD146	155Gd	P1H12	Fluidigm	3155006B
CD147	153Eu	HIM6	BD*	555961
CD172 a/b	163Dy	SE5A5	Fluidigm	3163017B
PDGFRa	160Gd	D13C6	Fluidigm	3160007A
CD184	175Lu	12G5	Fluidigm	3175001B
LAP (TGFβ1)	154Sm	TW4‐6H10	Biolegend*	349702

* Conjugation to the metal tag was performed by AbLab (www.ablab.ca).

### Adipogenic and osteogenic differentiation

Semiconfluent cultures were shifted from growth medium to adipogenic or osteogenic medium. The adipogenic medium (DMEM 1 g/L glucose, 10% FBS, 0.5 mmol/L isobutyl‐methylxanthine (Sigma), 60 μM indomethacin (Sigma), 10^−6^ M dexamethasone (Merck) and 5 μg/mL insulin.) was replaced every 3–4 days for 21 days. The cells were then fixed for 15 min in Baker's buffer and stained with Oil red O for 15 min at 37°C. The osteogenic medium [DMEM 4.5 g/L glucose, 10% FBS, 10^−7^ M dexamethasone, 50 μg/mL ascorbic acid (Sigma), 3 mM NaH_2_PO_4_ (Sigma)] was replaced every 3–4 days for 14 days. The cells were fixed for 20 min in 70% ethanol at −20°C and stained with Alizarin red for 45 min at RT.

### Immunofluorescence

Immunostaining was performed on 5 μm‐thick muscle cryosections, fixed in 4% PFA and incubated with PBS containing 2% FBS for 30 min or on fixed cells blocked with PBS 2% FBS 0.2% Triton. Cryosections or fixed cells were incubated for 1 h with primary antibodies (*Table* 3). After three washes in PBS, immune complexes were detected by incubation with the appropriate Alexa Fluor‐conjugated secondary antibodies purchased from Life Technologies at RT for 45 min. Nuclei and actin filaments were stained with Hoechst and phalloidin‐Alexa568 (Interchim), respectively.

**Table 3:** Antibodies used for immunofluorescence

Antigen	Host	Clone	Company	Cat#	Dilution
Lamin A/C	Mouse (IgG2b)	636	Novocastra	NCL‐Lam‐AC	1/400
Collagen type 7a1	Mouse (IgG1)	LH7.2	Sigma‐Aldrich	C6805	1/800
Desmin	Mouse (IgG1)	D33	Dako	M0760	1/50
Fibronectin	Mouse (IgG1)	568	Novocastra	NCL‐FIB	1/100
Laminin	Rabbit		Dako	Z0097	1/400
pSmad3	Rabbit	EP823Y	Abcam	Ab52903	1/150
Dystrophin 1	Mouse (IgG2a)	Dy4/6D3	Novocastra	NCL‐Dys1	1/20
Fibrillin 2	Rabbit		Invitrogen	PA5‐52995	1/100

### Quantitative PCR

Quantitative polymerase chain reaction (qPCR) was carried out using SYBR Green Mastermix (Roche Applied Science) in a LightCycler 480 Real‐Time PCR System (Roche Applied Science) with the following cycling protocol: 8 min at 95°C; followed by 50 cycles at 95°C for 15 s (s), 60°C for 15 s and 72°C for 15 s, and a final step consisting of 5 s at 95°C and 1 min at 65°C. The specificity of the PCR product was evaluated by melting curve analysis using the following program: 65°C to 97°C with a 0.11°C/s increase. Gene expression levels were normalized to h*RPLP0* or h*B2M* expression and quantified with the 2^–∆∆Ct^ method. The primer sequences are listed in *Table* 4.

**Table 4:** Primers used

	Forward	Reverse
TGF‐1	CGCGTGCTAATGGTGGAAAC	GTTCAGGTACCGCTTCTCGG
EDNRb	ATCTGCTTGCTTCATCCCGT	AAAGCAATCTGCATGCCACT
COL7A1	CCTCTGGTCCCCCTGGATTA	GGCGCCTGAATCTCCTTTCT
FBN2	ACCTCAACAGATGGCTCTCG	GCAGCACTGCATTTTCGTCA
MATN2	TCTGCACCCCAATGGCAAAAC	TGTTCAGACACAGCTGCTCAC
RplP0	TGGTCATCCAGCAGGTGTTCGA	ACAGACACTGGCAACATTGCGG
B2M	CTCTCTTTCTGGCCTGGAGG	TGCTGGATGACGTGAGTAAACC

### Live imaging

Live imaging was carried out on M^CT^, FibM^CT^, and FibM^OP^ CD56− cells cultured in growth medium and maintained at 37°C and 5% CO_2_ using a Nikon Ti microscope equipped with a plate specific chamber (Okolab), a CoolSNAP HQ2 camera (Roper Scientific) and an XY‐motorized stage (Nikon) driven by MetaMorph (Molecular Devices). We used the SkyPad algorithm to quantify live‐cell speed and distance. All data were generated from random fields and quantified blinded.

### Statistical analysis

Data are expressed as the mean ± SD. Statistical significance was assessed by ordinary or RM one‐way ANOVA followed by Tukey's multiple comparisons test. All statistical analyses were performed using GraphPad Prism (version 6.0d, GraphPad Software Inc.). Difference were considered to be significant at *P < 0.05, **P < 0.01, ***P < 0.001, and ****P < 0.0001.

## Results

### Increased fibrosis in human cricopharyngeal muscle

**Figure 1:**
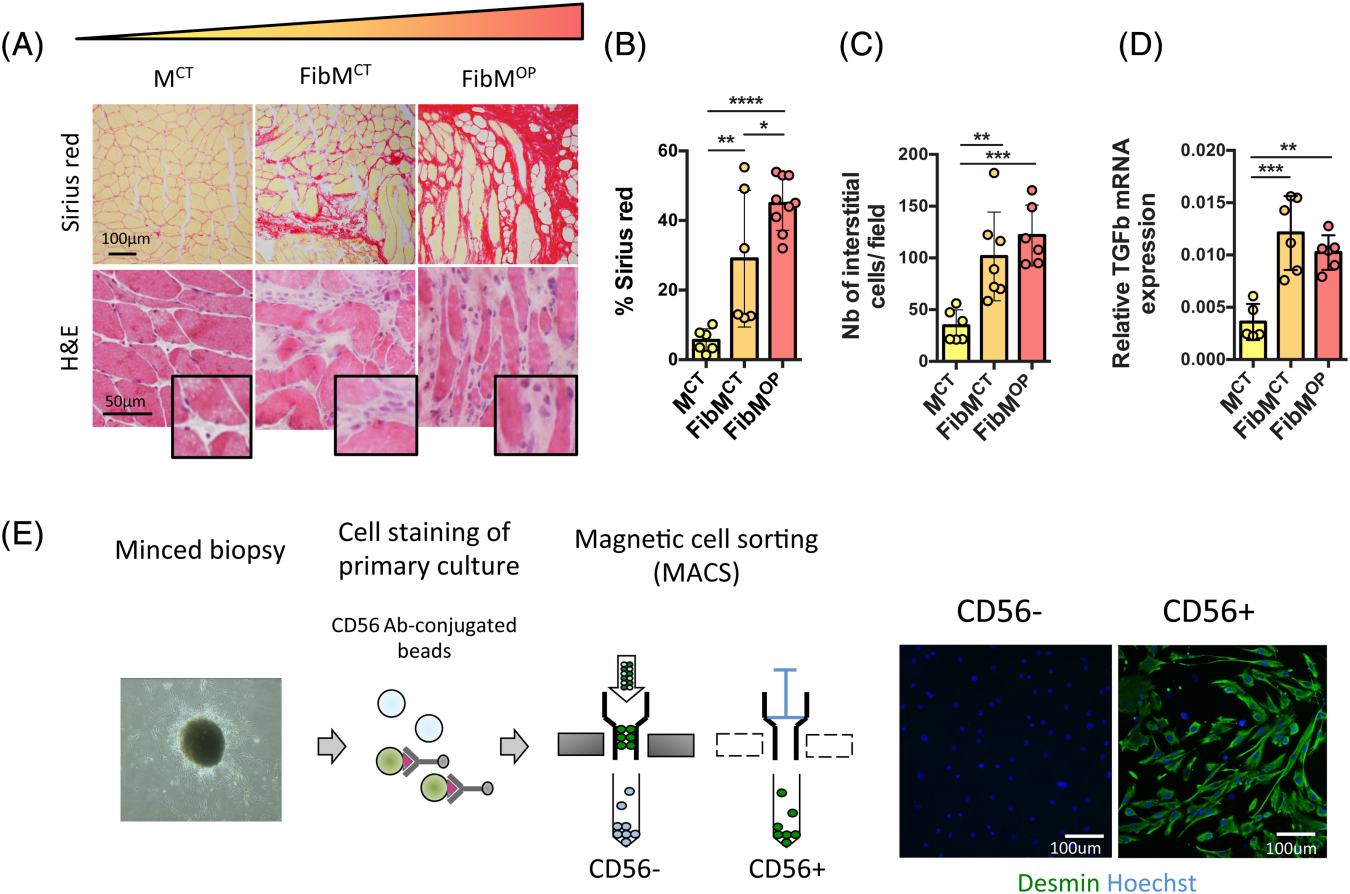
Isolation of nonmyogenic cells from nonfibrotic muscles (M^CT^) and muscles with physiological (FibM^CT^) and pathological (FibM^OP^) fibrosis. (*A*) Cross sections of human muscle biopsies stained with Sirius red to visualize and quantify fibrosis or haematoxylin and eosin (H&E). M^CT^, control muscle; FibM^CT^, fibrotic control muscle; FibM^OP^, fibrotic OPMD muscle. (*B*) Quantification of fibrosis by Sirius red staining of M^CT^, FibM^CT^ and FibM^OP^ human biopsies (*n* = 6–9 biopsies per condition). (*C*) Quantification of the number of interstitial cells (*n* = 6–7 muscle biopsies per condition). (*D*) RT‐qPCR quantification of *TGFβ* gene expression normalized to *RPLP0* expression in M^CT^, FibM^CT^, and FibM^OP^ human biopsies (*n* = 5–6 biopsies per condition). (*E*) Experimental scheme used for MACS isolation of nonmyogenic cells (CD56− cells) and myogenic cells (CD56+ cells) from human muscle biopsies. The number of myogenic cells in each population was assessed by desmin staining (green). Data are presented as means ± SD, with *P*‐values obtained by ordinary one‐way ANOVA test followed by Tukey's multiple comparisons test (*P < 0.05, **P < 0.01, ***P < 0.001, ****P < 0.0001).

To investigate the role of resident cell populations in human fibrosis, we isolated the nonmyogenic cell fractions from nonfibrotic and fibrotic (cricopharyngeal, CPM) human muscle biopsies (*Table* 1). We selected the CPM muscle because we have shown that it contains a large amount of connective tissue in healthy aged control subjects that is further increased in the pathological context of OPMD.^9^ This allowed us to compare nonmyogenic cells from nonpathological nonfibrotic muscle (M^CT^) to those from the nonpathological fibrotic CPM (FibM^CT^) and fibrotic CPM from patients with OPMD (FibM^OP^) as examples of physiological and pathological fibrotic muscle, respectively. By Sirius red and haematoxylin/eosin staining, we confirmed a high level of fibrosis and large number of interstitial cells in FibM^CT^ and FibM^OP^ muscle biopsies, with 30% (FibM^CT^) to 45% (FibM^OP^) connective tissue compared to 5% in M^CT^ (*Figure* 1A–C). This difference was accompanied by a higher expression level of transforming growth factor beta (TGFβ, the major profibrotic cytokine in dystrophic muscle^1^) in both FibM^CT^ and FibM^OP^ (*Figure* 1D).

### CyTOF analysis reveals a specific marker profile in FAPs from fibrotic muscle

**Figure 2:**
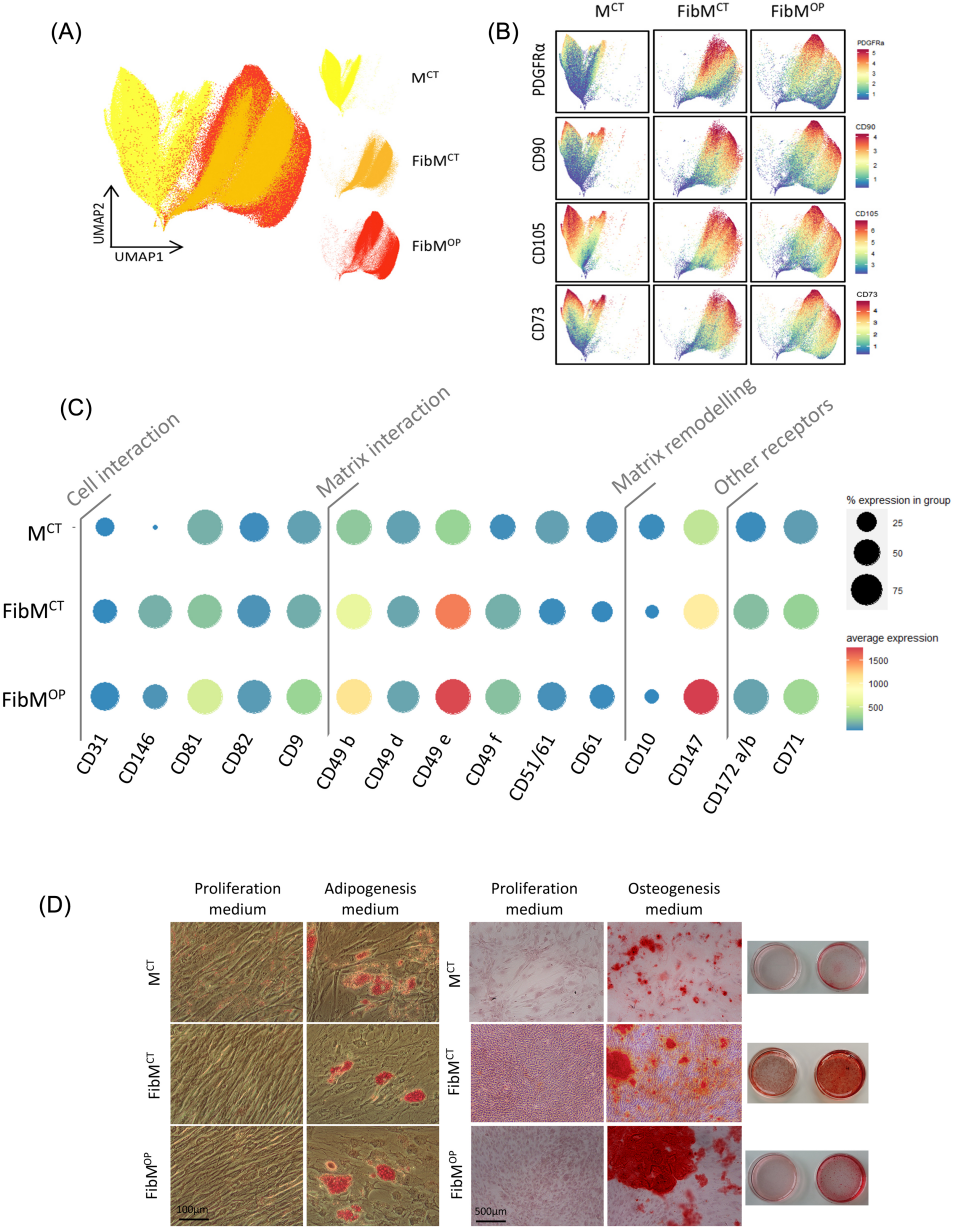
Human FAPs isolated from fibrotic muscles differ from those isolated from nonfibrotic muscles. (*A*) Uniform manifold approximation and projection (UMAP) map showing the distributions of cells from M^CT^ (yellow), FibM^CT^ (orange), and FibM^OP^ (red) muscle biopsies. For each condition, nonmyogenic cells were extracted, and data from three to four patients were concatenated. Each dot represents a single cell, and 300 000 cells were used to obtain the map. The 37 markers used for the analysis are listed in *Table* 2. (*B*) UMAP plots showing the expression patterns of PDGFRα, CD90, CD105, and CD73 in nonmyogenic cells from M^CT^, FibM^CT^, and FibM^OP^ muscle biopsies. Cells are coloured according to the intensity of the marker shown. (*C*) Expression dot plot of selected markers from the CyTOF analysis of nonmyogenic cells from fibrotic and nonfibrotic muscles. Dots are coloured according to the average intensity with which the marker was expressed, and the size of each dot shows the percentage of nonmyogenic cells expressing each marker in each condition: M^CT^, FibM^CT^, and FibM^OP^. (*D*) Adipogenic and osteogenic differentiation of human FAPs isolated from fibrotic or nonfibrotic muscles. Left panel: Oil red O staining of FAPs from M^CT^, FibM^CT^, and FibM^OP^ muscle biopsies in adipogenic differentiation medium. Right panel: Alizarin red staining of FAP cells from M^CT^, FibM^CT^, and FibM^OP^ muscle biopsies in osteogenic differentiation medium.

In order to determine surface markers that could uniquely distinguish fibrotic non‐muscle resident cells from nonfibrotic ones, we isolated primary cultures from M^CT^, FibM^CT^, and FibM^OP^ human muscle biopsies and separated the CD56− from the CD56+ fraction by MACS (*Figure* 1E). We then characterized the expression of markers in the nonmyogenic cells (CD56−) by cytometry by time of flight (CyTOF) using a panel of 37 markers, including markers of matrix remodelling, cell adhesion, and matrix interaction (*Table* 2). Using the UMAP algorithm to visualize the whole population, we observed that nonmyogenic cells from fibrotic (FibM^CT^ and FibM^OP^) and nonfibrotic (M^CT^) muscle plotted separately. However, each condition seemed to cluster together, showing the relative homogeneity of the populations from the three different types of muscle (*Figure* 2A). All of these cells expressed predominantly PDGFRα, CD90, CD105, and CD73, known markers of FAPs in human muscle^12^, ^14^, ^23^ (*Figures* 2B and S1), demonstrating that under our culture conditions, nonmyogenic cells isolated from human muscle are predominantly composed of FAPs. Interestingly, PDGFRβ and VCAM1 markers, which are expressed by FAPs in mdx mice, a mouse model of DMD, were also present in a large fraction of these cells, but the proportion of VCAM1+ cells was higher in the FibM^CT^ and FibM^OP^ cells than in the M^CT^ cells (*Figure* S2a,b). The accumulation of this injury‐activated subpopulation in mice was suggested to be associated with altered cross‐talk between inflammatory cells and FAPs, leading to the misclearance of profibrotic FAPs during muscle regeneration.^11^ A dot plot showing marker expression revealed that FAPs from fibrotic muscles (FibM^CT^ and FibM^OP^) expressed higher levels of cell adhesion molecules and integrin receptors, which facilitate matrix interaction, as well as matrix remodelling markers (*Figure* 2C). Interestingly, we found in fibrotic FAPs an increased expression of CD147 (aka EMMPRIN, extra‐cellular matrix metalloproteinase inducer) known to regulate migration, proliferation an invasion in cancer cells, but also to modulate MMP expression in a variety of physiological and pathological processes, including fibrosis.^24^ To confirm the identity of the cells as FAPs, we verified that these cells, from all three types of muscles, displayed adipogenic and osteogenic potential *in vitro* (*Figure* 2D). Together, these data suggest that under our culture conditions, CD56− nonmyogenic cells isolated from human muscle are FAPs (and are therefore referred to as FAPs hereafter), and that the FAPs from fibrotic muscles and those from control nonfibrotic muscles present different surface marker profiles.

### FAPs from fibrotic muscles present a high proliferative capacity and can impair muscle fusion

**Figure 3:**
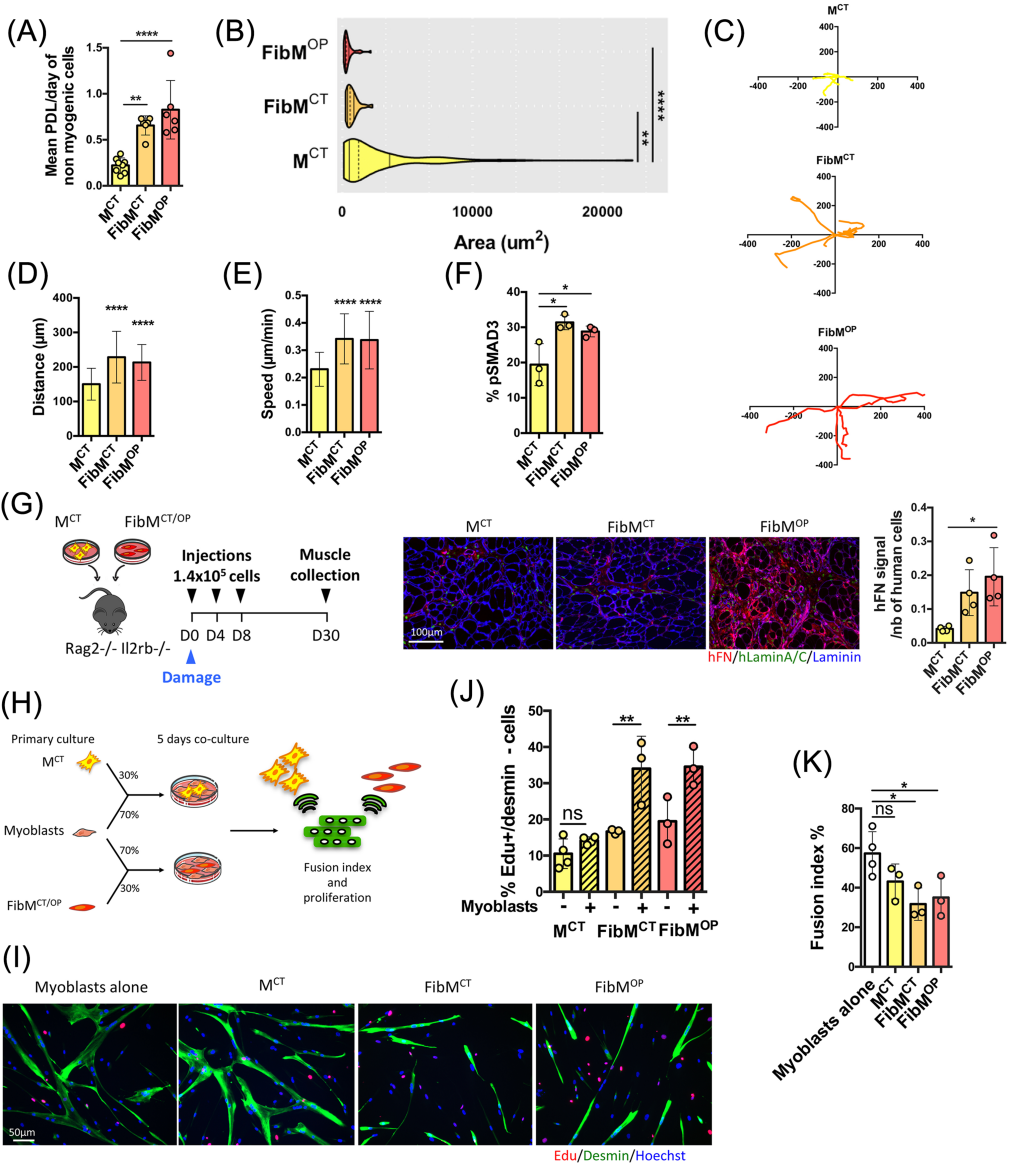
FAPs from fibrotic muscles have a higher proliferative capacity and a negative effect on fusion compared to those from nonfibrotic muscle. (*A*) Histogram showing the proliferation rate as the mean population doubling (PDL)/day for nonmyogenic cells isolated from M^CT^, FibM^CT^, and FibM^OP^ muscle biopsies over time (*n* = 6–8 biological replicates). (*B*) Violin plot representing the areas of nonmyogenic cells from M^CT^, FibM^CT^ and FibM^OP^ (*n* = 26–112 cells per condition). (*C*) Representative trajectory of cells over 24 h, (*D*) distance covered over 12 h (*n* = 25–39 cells per biological replicate; *n* = 3 per condition), and (*E*) speed of motion over 12 h evaluated by manual tracking using the SkyPad algorithm on live‐imaging videos of nonmyogenic cells from M^CT^, FibM^CT^, and FibM^OP^ (*n* = 25–39 cells per biological replicate; *n* = 3 per condition). (*F*) Percentage of basal pSMAD3+ cells assessed by immunostaining of serum‐starved FAPs from M^CT^, FibM^CT^, and FibM^OP^ muscle biopsies (*n* = 3 biological replicates). (*G*) Experimental scheme used to inject FAPs isolated from M^CT^, FibM^CT^, and FibM^OP^ muscle biopsies into the regenerating TA muscle of immunodeficient mice. A total of 1.4 × 10^5^ cells were injected at D0 after cryodamage and at D4 and D8 (left). Immunofluorescence analysis of cryosections was carried out using a human‐specific Lamin A/C antibody (hlaminA/C, green), a human‐specific fibronectin (hFN,red) antibody and a pan‐laminin antibody (blue) (middle). Quantification of ECM secretion as a ratio of hFN signal divided by the number of human cells (*n* = 4 per condition) (right). (*H*) Experimental scheme of the coculture experiments. FAPs from nonfibrotic and fibrotic muscles were cocultured with myoblasts at a 30%/70% ratio for 5 days in differentiation medium. Proliferation and the fusion index were assessed to evaluate the cross‐talk between nonmyogenic and myogenic cells. (*I*) Representative coimmunostaining of desmin (green) and EdU (red) after 5 days of coculture of FAPs and myogenic cells. Nuclei were counterstained with Hoechst (blue). (*J*) Quantification of EdU incorporation in FAPs from M^CT^, FibM^CT^, and FibM^OP^ alone or in coculture with myotubes (hatched bars) after 5 days in differentiation medium (*n* = 3–4 biological replicates; ns = nonsignificant). (*K*) the fusion index after 5 days of differentiation was assessed by Desmin staining in myogenic cells alone and in coculture with 30% M^CT^, FibM^CT^, or FibM^OP^ FAPs (*n* = 3–4 biological replicates). Data are presented as means ± SD, with *P*‐values obtained by ordinary or RM one‐way ANOVA test followed by Tukeys multiple comparisons test (*P < 0.05, **P < 0.01, ***P < 0.001, ****P < 0.0001).

The differences in the marker profiles of fibrotic and nonfibrotic cells prompted us to investigate whether their behaviour would also differ *in vitro*. To test this hypothesis, we cultured FAPs isolated from FibM^CT^, FibM^OP^, and M^CT^ muscle in proliferation conditions. We observed a much higher proliferation rate in cells isolated from FibM^CT^ and FibM^OP^ muscle biopsies compared to those isolated from M^CT^ muscle biopsies (*Figure* 3A). In addition to their greatly enhanced proliferative capacity, live‐cell imaging revealed that cells from fibrotic muscles (FibM^CT^ and FibM^OP^) were much more motile and smaller than those from non‐fibrotic muscle (*Figures* 3B–E and S3a). Because TGFβ is a key profibrogenic cytokine in muscle^1^ that was found to be up‐regulated in our fibrotic muscles (*Figure* 1C), we investigated whether the TGFβ signalling was active in FAPs isolated from fibrotic muscles. TGFβ binds to a TGFβ‐receptor complex that will phosphorylate SMAD2/3, which will subsequently accumulate in the nucleus and act as transcription factors. We therefore assessed the localization of the phosphorylated SMAD2/3 (p‐SMAD2/3) proteins under serum‐starved conditions and identified an elevated basal level of p‐SMAD2/3 in FibM^CT^ and FibM^OP^ FAPs compared with that of M^CT^ (*Figures* 3F and S3b), showing that the canonical TGFβ‐SMAD pathway is active in FibM^CT^ and FibM^OP^ FAPs. In contrast, the activity of ERK1/2 was not increased in FibM^OP^ cells (data not shown) compared with M^CT^ cells suggesting activation of the canonical TGFβ pathway but not the non‐canonical pathway. When FAPs were injected *in vivo* in the regenerating muscle of immunodeficient mice, the ECM secretion was also increased in FibM^CT^ and FibM^OP^ FAPs compared to M^CT^ FAPs (*Figure* 3G).

We next evaluated the proliferation of the cells when cocultured with myogenic cells to mimic *in vivo* interactions, through which FAPs and muscle cells communicate with each other. Muscle cells and FAPs were seeded together at a 70%/30% ratio (*Figure* 3H). Once the cells adhered, muscle cell differentiation was induced for 5 days in the presence of 10 μM EdU (*Figure* 3I). We observed an increased percentage of EdU‐positive FibM^CT^ and FibM^OP^ FAPs compared to EdU‐positive M^CT^ FAPs, indicating an increased proliferation in the presence of myotubes (*Figure* 3J). The known secretion of TGFβ by myotubes^25^ and the observed activation of SMAD2/3 in FAPs (*Figures* 3F and S3b), suggest that this effect on proliferation might be a consequence of TGFβ signalling. Interestingly, using desmin immunostaining to label myogenic cells, we also observed an impaired fusion in the presence of FibM^CT^ and FibM^OP^ FAPs, whereas M^CT^ FAPs had no effect on cell fusion (*Figure* 3I,K). In addition, as FibM^CT^ and FibM^OP^ FAPs are much smaller than M^CT^ FAPs (*Figure* 3B), this impaired fusion was not due to large cells blocking the fusion process. Together, these data suggest that cross‐talk occurs between fibrotic cells and muscle fibres, leading to the hyperproliferation of fibrotic cells and impaired muscle fusion.

### The endothelin receptor is a new targetable regulator of fibrosis

**Figure 4:**
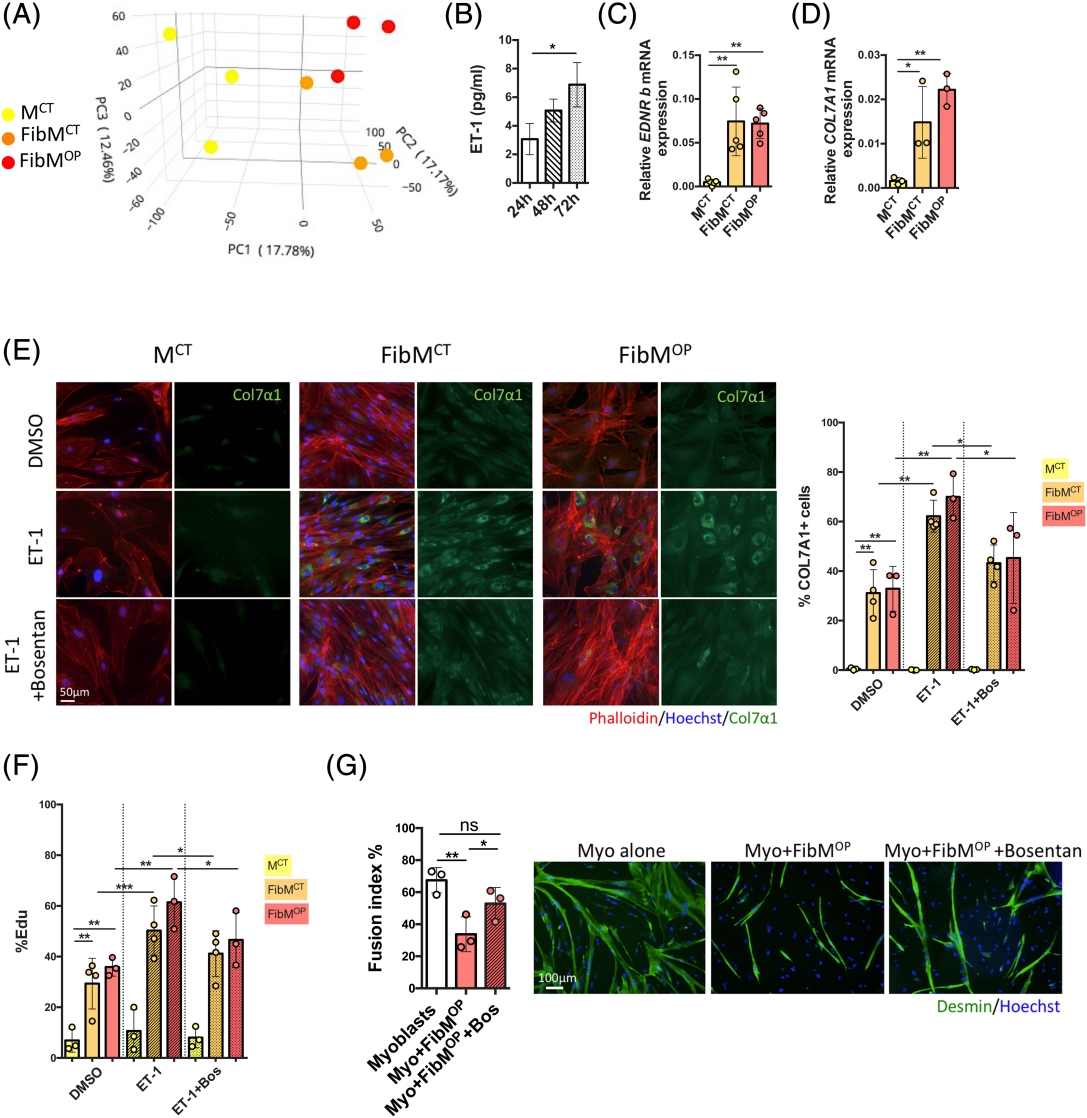
Targeting ET‐1‐mediated secretion of fibrotic FAPs improves myoblast fusion. (*A*) Principal component analysis (PCA) plot prepared following transcriptome analysis of FAPs from M^CT^ (yellow), FibM^CT^ (orange), and FibM^OP^ (red) muscle biopsies. Each dot represents cells from one patient. (*B*) Quantification of ET‐1 protein levels in unconcentrated conditioned medium from differentiated human myoblasts after 24, 48, and 72 h of differentiation, reanalyzed from a previous study.^26^ (*C*) RT‐qPCR quantification of *EDNRb* gene expression normalized to *RPLP0* expression in FAPs from M^CT^, FibM^CT^, and FibM^OP^ muscle biopsies (*n* = 5 biological replicates). (*D*) RT‐qPCR quantification of *Col7a1* gene expression normalized to *RPLP0* expression in FAPs from M^CT^, FibM^CT^, and FibM^OP^ muscle biopsies (*n* = 3 biological replicates). (*E*) FAPs from M^CT^, FibM^CT^, and FibM^OP^ muscle biopsies were cultured in the presence of ET‐1 (40 nM) or ET‐1 and bosentan (10 μM) for 3 days in proliferation medium containing 1% FBS. Left: Immunofluorescence analysis of Phalloidin (red), Hoechst (blue) and collagen 7a1 (green) was performed. Right: Quantification of the percentage of COL7A1‐positive cells (*n* = 3–4 biological replicates). (*F*) Quantification of the percentage of EdU incorporation after treatment of FAPs from M^CT^, FibM^CT^, and FibM^OP^ muscle biopsies with ET‐1 (40 nM) or ET‐1 and bosentan (10 μM) for 3 days in proliferation medium containing 1% FBS (*n* = 3–4 biological replicates). (*G*) Myoblasts were cocultured alone or with FAPs from fibrotic muscles at a 70/30% ratio for 5 days in differentiation medium. Bosentan (10 μM) was added on days 0 and 3 of differentiation. Left: The fusion index was assessed by Desmin staining (*n* = 3 biological replicates). Right: Desmin immunostaining and Hoechst staining (scale bar = 100 μm). Data are presented as means ± SD, with *P*‐values obtained by ordinary or RM one‐way ANOVA test followed by Tukey's multiple comparisons test (*P < 0.05, **P < 0.01, ***P < 0.001, ****P < 0.0001).

To identify factors which could lead to this hyperproliferation and consequently provide new anti‐fibrotic targets, we analysed the transcriptomes of proliferating cells isolated from nonfibrotic M^CT^ muscles and fibrotic FibM^CT^ and FibM^OP^ muscles. Principal component analysis (PCA) of the transcriptome data confirmed the results of CyTOF: FAPs from fibrotic muscles expressed a different set of genes than those from control non‐fibrotic muscle (*Figures* 4A and S3c). To identify potential targetable hits expressed by FAPs from fibrotic muscles (but not by FAPs from nonfibrotic muscles), we examined the transcriptome data to identify cellular receptors up‐regulated under fibrotic conditions but not in control conditions (Table S1a,b). Among the receptors, we identified endothelin receptor type B (EDNRB) to be a particularly interesting candidate since endothelin‐1 (ET‐1), its ligand, is known to be a profibrotic peptide and ET‐1 is secreted by human myotubes (*Figure* 4B, ^26^). In addition, because our previous experiment (*Figure* 3I–K) showed a cross‐talk between fibrotic FAPs and muscle fibres, we used the transcriptome data to perform *in silico* computational secretome analysis to identify ECM components potentially secreted by fibrotic FAPs. We subjected the 118 and 116 genes with up‐regulated expression in the FibM^CT^ and FibM^OP^ FAPs compared with the M^CT^ FAPs to computational filtering to predict their cellular localization and identify the proteins most likely to be secreted into the extracellular space^22^ (Table S1a,b). Among the extracellular protein candidates whose expression was up‐regulated in FAPs from both FibM^CT^ and FibM^OP^ FAPs, compared with control FAPs, we found the ECM components COL7A1, MATN2, and FBN2. Using qPCR, we confirmed the increased expression of *EDNRB*, *COL7A1*, *MATN2*, and *FBN2* mRNA in fibrotic FAPs compared to M^CT^ FAPs (*Figures* 4C,D and S4a). By immunostaining, we also confirmed increased COL7A1 protein levels in human fibrotic muscle sections compared with nonfibrotic muscle sections (*Figure* S4b). We also confirmed that FibM^CT^ and FibM^OP^ FAPs secrete more COL7A1 than control cells *in vitro* (*Figure* 4E) and more COL7A1 and FBN2 *in vivo* (*Figure* S4c).

We further confirmed the potential involvement of ET‐1 by investigating the effect of exogenous ET‐1 treatment on FAPs from the three different types of muscle. FAPs at 70–80% confluence were treated for 3 days in 1% FBS‐containing medium with either DMSO or 100 ng/mL ET‐1. Treatment with ET‐1 increased COL7A1 protein production in fibrotic FAPs but not in control FAPs, while the addition of 10 μM bosentan, an EDNR antagonist,^27^ abolished the effect of ET‐1, confirming that the pathway downstream of this receptor is involved in ECM hypersecretion (*Figure* 4E). Using EdU, we demonstrated that 100 ng/mL ET‐1 treatment increased the proliferation of fibrotic FAPs, whereas it had no effect on M^CT^ FAPs. This effect was also prevented by the EDNR antagonist bosentan (*Figure* 4F). Because coculture of FAPs from fibrotic muscle impaired muscle fusion (*Figure* 3I–K), we performed the same coculture experiment with muscle cells and FAPs at a 70%/30% ratio in the presence of bosentan. Differentiation was then induced for 5 days. Using desmin immunostaining, we found that the impaired fusion observed upon coculture with FibM^OP^ FAPs was partially restored by blocking the endothelin receptor EDNRB (*Figure* 4G), while no effect of bosentan on fusion was observed in myoblasts alone (*Figure* S5a). Similarly, Bosentan alone on FAPs had no effect on COL7A1 secretion or proliferation (*Figure* S5b). Together, our results demonstrate a key role for endothelin in human muscle fibrosis and pinpoint EDNRB as a new therapeutic target to counteract human fibrosis.

## Discussion

Although fibrosis is one of the most detrimental complications of trauma, muscle aging, and muscular dystrophies, the vast majority of our knowledge on fibrosis originates from studies in murine models of muscular dystrophies. However, both the degenerative process and fibrosis differ between mice and humans, which prompted us to investigate the cellular processes involved in fibrosis using human samples. In the present study, we isolated nonmyogenic cells from human muscles under non‐fibrotic and fibrotic conditions and cocultured the cells to characterize them regarding proliferation and secretion.

TGFβ is the best known trigger of fibrosis.^28^ In fibrotic muscle from dystrophic mice, TGFβ drives differentiation of FAPs into matrix‐producing cells.^15^ TGFβ is released by muscle fibres and macrophages in its latent form, in association with latent‐associated peptide (LAP). When released, the inactive LAP‐TGFβ complex must be activated either enzymatically or mechanically to exert its pro‐fibrotic effects.^28^ We observed high TGFβ1 expression in our human fibrotic muscles and activation of the canonical TGFβ‐SMAD pathway (and not the noncanonical ERK1/2 pathway,^29^ data not shown) in FAPs isolated from fibrotic muscle. Interestingly, in our study, CyTOF analysis of fibrotic samples also showed that human FAPs expressed LAP‐bound TGFβ1 (*Figure* S2c). Although macrophages, which produce TGFβ,^15^, ^18^, ^30^ were absent in our coculture system, we observed both the increased proliferation of FAPs and impaired fusion, suggesting that in addition to muscle fibres and macrophages, FAPs themselves also produce TGFβ1. Further studies are needed to elucidate the respective contributions of muscle fibres, macrophages, and FAPs to the TGFβ pathway. Considering that the TGFβ pathway inhibits myoblast fusion,^25^, ^31^ we propose that in addition to the secretion of ECM components, TGFβ produced by FAPs themselves also has a direct negative effect on muscle differentiation.

TGFβ has been well characterized as a potent cell growth factor that is involved in promoting the development of fibrosis.^1^ However, inhibitors of TGFβ, such as neutralizing antibodies, have off‐target effects,^32^ and therapeutic attempts to target this pathway have been rather unsuccessful, suggesting that the human fibrotic process is more complex and that other targets are involved. With the goal of identifying druggable targets other than TGFβ, we analysed the transcriptomes of fibrotic and non‐fibrotic FAPs and found that FAPs from fibrotic muscle (from both healthy and OPMD individuals), but not those from nonfibrotic muscle from healthy individuals, express EDNRB, a receptor for endothelin. We also demonstrated that ET‐1 is produced by myotubes, and the expression of EDNRB was increased in the FAPs isolated from fibrotic muscle compared to those isolated from nonfibrotic muscle. ET‐1 has a well‐known role in the cardiovascular system^33^ and in fibrosis in organs other than skeletal muscle and diseases other than dystrophies.^34^ Interestingly, we observed that in the presence of bosentan, an EDNRB antagonist, the production of ECM components by fibrotic FAPs such as COL7A1, and FAPs proliferation were decreased. Collagen VII is the main component of anchoring fibrils which connects the external epithelia to the underlying stroma in skin. TGFβ has been shown to activate the COL7A1 promoter in a SMAD dependent manner.^35^ To our knowledge, Collagen VII expression has not been studied in skeletal muscle, but its involvement in tissue remodelling has been suggested,^36^ and we found it expressed by murine FAPs during muscle regeneration in published single‐cell analysis.^37^ We also observed that bosentan treatment partially rescued the negative effects of FAPs on muscle differentiation. Taken together, these results suggest that ET‐1 is partly responsible for the impaired fusion observed *in vitro*; thus, antagonists of EDNRB, such as bosentan, may be potential drugs which could be used to combat fibrosis in muscle.

Our results also reveal a privileged crosstalk between muscle fibres and fibrotic FAPs: we showed that muscle fibres produce ET‐1, that fibrotic FAPs overexpress its receptor EDNRB, that ET‐1 addition maintains FAPs in a prosecretory state, and that this overall combination leads to a negative feedback loop with both impaired muscle differentiation and increased ECM secretion. ET‐1 has been also suggested to promote senescence,^38^ but in our conditions, there was no evidence of increased senescence in FAPs in response to ET‐1 as determined by B‐gal activity assay and p16 and p21 RNA expression (data not shown). Altogether, these data suggest that in a muscle environment TGFβ and ET‐1 may both trigger a profibrotic phenotypic switch in FAPs, leading to their increased proliferation and ECM secretion.

The data collected in this study showed that in physiological (high level of ECM in control CPM) and pathological (excessive ECM deposition in OPMD CPM) human muscle fibrosis, the populations of FAPs involved were globally similar, indicating that TGFβ and ET‐1 are broadly implicated in human muscle fibrosis. It is known that increase in connective tissue can eventually lead to dysphagia in the elderly: dysphagia is known to affect about 15–30% of aged individuals.^39^ In our observations, this connective tissue is increased in OPMD patients, and consequently, the fibrosis would have a negative effect causing a stiffening of the tissue and problems in deglutition. Further studies are needed to decipher specific changes in muscle‐associated fibrosis in pathological conditions like OPMD (Table S2). Together, our results suggest that muscle requires the coordinated and well‐orchestrated interaction between muscle fibres and FAPs, and that any alteration in this interaction can perturb the overall muscle function, and lead to the establishment of a pathological phenotype in the FAPs. Our data suggest that TGFβ and ET‐1 blockade might represent a potential future strategy to treat muscle fibrosis, particularly in the pharyngeal muscles of patients suffering from OPMD and achalasia but also in other muscle pathologies involving fibrosis. This is important since many of the more recent innovative therapies, either gene or cell based show a reduced efficacy due to the fibrotic nature of the targeted muscle.

## Conflict of interest

M.B., L.M., A.B., E.N., and C.T. are inventors on a patent on targeting endothelin to treat fibrosis. A.Bo., L.G., V.A., V.Y., J.D., A.O., V.H., T.G., S.P., J.L.S.G., A.C., V.M., and G.B.B. have declared that no conflicts of interest exist.

## Supporting information




**Table S1a** Table of Upregulated genes in FibMct FAPs vs Mct FAPs

**Table S1b** Table of Upregulated genes in FibMop FAPs vs Mct FAPs

**Table S2** Table of differentially expressed genes in FibMop FAPs vs FibMct FAPs

**Fig S1** Expression of FAP markers by CyTOF.
Mass cytometry contour plots coloured by density showing the gating strategy for the PDGFRα, CD90 and CD105 markers for each individual from which M^CT^, FibM^CT^ and FibM^OP^ muscle biopsies were obtained.
**Fig** **S2** CD106, PDGFRβ and LAP (TGFβ1) expression in FAP cells from fibrotic and nonfibrotic muscles by CyTOF.
UMAP plots showing the expression patterns of (a) CD106 (VCAM1), (b) PDGFRβ and (c) LAP (TGFβ1) in nonmyogenic cells from M^CT^ FibM^CT^ and FibM^OP^ muscle biopsies. The cells are coloured according to the intensity with which the marker was expressed. For CD106 and PDGFRβ the gating strategy for each patient is highlighted. A histogram recapitulating those percentages under the different conditions is also shown (***P < 0.001, ****P < 0.0001).
**Fig S3** Comparison of CD56‐ cells from fibrotic and nonfibrotic muscles.
(a) Live imaging video of CD56‐ cells from M^CT^, FibM^CT^ and FibM^OP^ muscle biopsies in growth medium with 20% serum. (b) Representative nuclear staining of basal pSMAD3 + expression on serum‐starved FAPs from M^CT^, FibM^CT^ and FibM^OP^ muscle biopsies (*n* = 3 biological replicates). (c) Additional view of the PCA presented in Figure 3 highlighting specifically PC1 versus PC2 with all cell types (left) and PC2 versus PC3 with FibM^CT^ and FibM^OP^ only (right). M^CT^ (yellow), FibM^CT^ (orange) and FibM^OP^ (red) muscle biopsies. Each dot represents cells from one patient.
**Fig** **S4** ECM secretion from nonfibrotic and fibrotic muscles.
(a) RT‐qPCR quantification of *FBN2* and *MATN2* gene expression normalized to *B2M* expression in FAPs from M^CT^, FibM^CT^ and FibM^OP^ muscle biopsies (*n* = 3 biological replicates). (b) Immunofluorescence analysis of Dystrophin (green), Hoechst (blue) and Collagen7a1 (red) was performed on non‐fibrotic (M^CT^) and fibrotic (FibM^OP^) muscles. (c) Experimental scheme used to inject FAPs isolated from M^CT^, FibM^CT^ and FibM^OP^ muscle biopsies into the regenerating TA muscle of immunodeficient mice. A total of 1.4 × 10^e^ cells were injected at D0 after cryodamage and at D4 and D8. Muscle were collected at D30. hCOL7A1 (top) and hFBN2 (bottom) immunostaining after the injection of FAPs isolated from M^CT^, FibM^CT^ and FibM^OP^ muscle biopsies into the regenerating TA muscle of immunodeficient mice. Cryosections were stained using a human‐specific lamin A/C antibody (hlaminA/C, green), a human‐specific collagen 7a1 (hCol7a1, red) antibody, a human‐specific fibrillin 2 antibody (hFBN2, red) and a pan‐laminin antibody (blue). Scale bar = 100 μm.
**Fig S5** Effect of Bosentan alone on myoblasts and FAPs
(a) Myoblasts were cultured for 5 days in differentiation medium. Bosentan (10 μM) was added on days 0 and 3 of differentiation. Fusion index was assessed by Desmin staining (*n* = 3 technical replicates). (b) FAPs from M^CT^, FibM^CT^ and FibM^OP^ were cultured in the presence of bosentan alone (10 μM) for 3 days in proliferation medium containing 1% FBS. Left: quantification of the percentage of COL7A1‐positive cells (*n* = 3 technical replicates of 1 biological sample for each condition). Right: quantification of the percentage of EdU incorporation after treatment of FAPs from M^CT^, FibM^CT^ and FibM^OP^ with bosentan alone (10 μM) for 3 days in proliferation medium containing 1% FBS (*n* = 3 technical replicates of 1 biological sample for each condition).

## References

[1] Smith LR , Barton ER . Regulation of fibrosis in muscular dystrophy. Matrix Biol 2018;68–69:602–615. 10.1016/j.matbio.2018.01.014 PMC6519730 29408413

[2] Abrigo J , Simon F , Cabrera D , Cordova G , Trollet C , Cabello‐Verrugio C . Central role of transforming growth factor type beta 1 in skeletal muscle dysfunctions: an update on therapeutic strategies. Curr Protein Pept Sci 2018;19:1189–1200. 29150918 10.2174/1389203718666171117101916

[3] Uezumi A , Fukada S , Yamamoto N , Takeda S , Tsuchida K . Mesenchymal progenitors distinct from satellite cells contribute to ectopic fat cell formation in skeletal muscle. Nat Cell Biol 2010;12:143–152. 20081842 10.1038/ncb2014

[4] Joe AWB , Yi L , Natarajan A , Le Grand F , So L , Wang J , et al. Muscle injury activates resident fibro/adipogenic progenitors that facilitate myogenesis. Nat Cell Biol 2010;12:153–163. 20081841 10.1038/ncb2015 PMC4580288

[5] Pessina P , Kharraz Y , Jardí M , Fukada S , Serrano AL , Perdiguero E , et al. Fibrogenic cell plasticity blunts tissue regeneration and aggravates muscular dystrophy. Stem Cell Reports 2015;4:1046–1060. 25981413 10.1016/j.stemcr.2015.04.007 PMC4472037

[6] Klinger W , Jurkat‐Rott K , Lehmann‐Horn F , Schleip R . The role of fibrosis in Duchenne muscular dystrophy. Acta Myol 2012;31:184–195. 23620650 PMC3631802

[7] Hogarth MW , Defour A , Lazarski C , Gallardo E , Manera JD , Partridge TA , et al. Fibroadipogenic progenitors are responsible for muscle loss in limb girdle muscular dystrophy 2B. Nat Commun 2019;10:2430. 31160583 10.1038/s41467-019-10438-z PMC6547715

[8] Desguerre I , Arnold L , Vignaud A , Cuvellier S , Yacoub‐youssef H , Gherardi RK , et al. A new model of experimental fibrosis in hindlimb skeletal muscle of adult mdx mouse mimicking muscular dystrophy. Muscle Nerve 2012;45:803–814. 22581532 10.1002/mus.23341

[9] Gidaro T , Negroni E , Perié S , Mirabella M , Lainé J , Lacau St Guily J , et al. Atrophy, fibrosis, and increased PAX7‐positive cells in pharyngeal muscles of oculopharyngeal muscular dystrophy patients. J Neuropathol Exp Neurol 2013;72:234–243. 23399899 10.1097/NEN.0b013e3182854c07

[10] Trollet C , Anvar SY , Venema A , Hargreaves IP , Foster K , Vignaud A , et al. Molecular and phenotypic characterization of a mouse model of oculopharyngeal muscular dystrophy reveals severe muscular atrophy restricted to fast glycolytic fibres. Hum Mol Genet 2010;19:2191–2207. 20207626 10.1093/hmg/ddq098

[11] Malecova B , Gatto S , Etxaniz U , Passafaro M , Cortez A , Nicoletti C , et al. Dynamics of cellular states of fibro‐adipogenic progenitors during myogenesis and muscular dystrophy. Nat Commun 2018;9:3670. 30202063 10.1038/s41467-018-06068-6 PMC6131350

[12] Madaro L , Passafaro M , Sala D , Etxaniz U , Lugarini F , Proietti D , et al. Denervation‐activated STAT3‐IL‐6 signalling in fibro‐adipogenic progenitors promotes myofibres atrophy and fibrosis. Nat Cell Biol 2018;20:917–927. 30050118 10.1038/s41556-018-0151-y PMC6145844

[13] Lukjanenko L , Karaz S , Stuelsatz P , Gurriaran‐Rodriguez U , Michaud J , Dammone G , et al. Aging disrupts muscle stem cell function by impairing matricellular WISP1 secretion from fibro‐adipogenic progenitors. Cell Stem Cell 2019;24:433, e7–446. 30686765 10.1016/j.stem.2018.12.014 PMC6408230

[14] Contreras O , Rossi FMV , Theret M . Origins, potency, and heterogeneity of skeletal muscle fibro‐adipogenic progenitors‐time for new definitions. Skelet Muscle 2021;11(1):16. 34210364 10.1186/s13395-021-00265-6 PMC8247239

[15] Juban G , Saclier M , Yacoub‐Youssef H , Kernou A , Arnold L , Boisson C , et al. AMPK activation regulates LTBP4‐dependent TGF‐β1 secretion by pro‐inflammatory macrophages and controls fibrosis in duchenne muscular dystrophy. Cell Rep 2018;25(8):2163–2176. 30463013 10.1016/j.celrep.2018.10.077

[16] Contreras O , Soliman H , Theret M , Rossi FMV , Brandan E . TGF‐β‐driven downregulation of the transcription factor TCF7L2 affects Wnt/β‐catenin signaling in PDGFRα+ fibroblasts. J Cell Sci 2020;133:jcs242297. 32434871 10.1242/jcs.242297

[17] Dulauroy S , Di Carlo SE , Langa F , Eberl G , Peduto L . Lineage tracing and genetic ablation of ADAM12+ perivascular cells identify a major source of profibrotic cells during acute tissue injury. Nat Med 2012;18:1262–1270. 22842476 10.1038/nm.2848

[18] Lemos DR , Babaeijandaghi F , Low M , Chang C‐K , Lee ST , Fiore D , et al. Nilotinib reduces muscle fibrosis in chronic muscle injury by promoting TNF‐mediated apoptosis of fibro/adipogenic progenitors. Nat Med 2015;21:786–794. 26053624 10.1038/nm.3869

[19] Mozzetta C , Consalvi S , Saccone V , Tierney M , Diamantini A , Mitchell KJ , et al. Fibroadipogenic progenitors mediate the ability of HDAC inhibitors to promote regeneration in dystrophic muscles of young, but not old Mdx mice. EMBO Mol Med 2013;5:626–639. 23505062 10.1002/emmm.201202096 PMC3628105

[20] Lacau St Guily J , Zhang KX , Périé , Copin H , Butler‐Browne GS , Barbet JP . Improvement of dysphagia following cricopharyngeal myotomy in a group of elderly patients. Histochemical and biochemical assessment of the cricopharyngeal muscle. Ann Otol Rhinol Laryngol 1995;104:603–609. 7639468 10.1177/000348949510400803

[21] Negroni E , Riederer I , Chaouch S , Belicchi M , Razini P , Di Santo J , et al. In vivo myogenic potential of human CD133+ muscle‐derived stem cells: a quantitative study. Mol Ther 2009;17(10):1771–1778. 19623164 10.1038/mt.2009.167 PMC2835017

[22] Zhao L , Poschmann G , Waldera‐Lupa D , Rafiee N , Kollmann M , Stühler K . OutCyte: a novel tool for predicting unconventional protein secretion. Sci Rep 2019;9:19448. 31857603 10.1038/s41598-019-55351-z PMC6923414

[23] Goloviznina NA , Xie N , Dandapat A , Iaizzo PA , Kyba M . Prospective isolation of human fibroadipogenic progenitors with CD73. Heliyon 2020;6:e04503. 32728644 10.1016/j.heliyon.2020.e04503 PMC7381701

[24] Xiong L , Edwards CK , Zhou L . The biological function and clinical utilization of CD147 in human diseases: a review of the current scientific literature. Int J Mol Sci 2014;15:17411–17441. 25268615 10.3390/ijms151017411 PMC4227170

[25] Girardi F , Taleb A , Ebrahimi M , Datye A , Gamage DG , Peccate C , et al. TGFβ signaling curbs cell fusion and muscle regeneration. Nat Commun 2021;12:750. 33531466 10.1038/s41467-020-20289-8 PMC7854756

[26] Le Bihan M‐C , Bigot A , Jensen SS , Dennis JL , Rogowska‐Wrzesinska A , Lainé J , et al. In‐depth analysis of the secretome identifies three major independent secretory pathways in differentiating human myoblasts. J Proteomics 2012;77:344–356. 23000592 10.1016/j.jprot.2012.09.008

[27] Clozel M , Breu V , Gray GA , Burri K , Hirth G , Müller M , et al. Pharmacological characterization of bosentan, a new potent orally active nonpeptide endothelin receptor antagonist. J Pharmacol Exp Therap 1994;270:228–235. 8035319

[28] Meng X , Nikolic‐Paterson DJ , Lan HY . TGF‐β: the master regulator of fibrosis. Nat Rev Nephrol 2016;12:325–338. 27108839 10.1038/nrneph.2016.48

[29] Clayton SW , Ban GI , Liu C , Serra R . Canonical and noncanonical TGF‐β signaling regulate fibrous tissue differentiation in the axial skeleton. Sci Rep 2020;10:21364. 33288795 10.1038/s41598-020-78206-4 PMC7721728

[30] Baht GS , Bareja A , Lee DE , Rao RR , Huang R , Huebner JL , et al. Meteorin‐like facilitates skeletal muscle repair through a Stat3/IGF‐1 mechanism. Nat Metab 2020;2:278–289. 32694780 10.1038/s42255-020-0184-y PMC7504545

[31] Melendez J , Sieiro D , Salgado D , Morin V , Dejardin M‐J , Zhou C , et al. TGFβ signalling acts as a molecular brake of myoblast fusion. Nat Commun 2021;12:749. 33531476 10.1038/s41467-020-20290-1 PMC7854724

[32] Varga J , Pasche B . Antitransforming growth factor‐β therapy in fibrosis: recent progress and implications for systemic sclerosis. Curr Opinion Rheumatol 2008;20(6):720–728. 10.1097/BOR.0b013e32830e48e8 PMC4541793 18946334

[33] Yanagisawa M , Kurihara H , Kimura S , Tomobe Y , Kobayashi M , Mitsui Y , et al. A novel potent vasoconstrictor peptide produced by vascular endothelial cells. Nature 1988;332:411–415. 2451132 10.1038/332411a0

[34] Angulo O . Endothelin‐1, A key player in sarcopenia? ECOrthopaedics 2019;82–85.

[35] Vindevoghel L , Kon A , Lechleider RJ , Uitto J , Roberts AB , Mauviel A . Smad‐dependent transcriptional activation of human type VII collagen gene (COL7A1) promoter by transforming growth factor‐beta. J Biol Chem 1998;273:13053–13057. 9582342 10.1074/jbc.273.21.13053

[36] Wessner B , Liebensteiner M , Nachbauer W , Csapo R . Age‐specific response of skeletal muscle extracellular matrix to acute resistance exercise: a pilot study. Eur J Sport Sci 2019;19:354–364. 30293527 10.1080/17461391.2018.1526974

[37] De Micheli AJ , Laurilliard EJ , Heinke CL , Ravichandran H , Fraczek P , Soueid‐Baumgarten S , et al. Single‐cell analysis of the muscle stem cell hierarchy identifies heterotypic communication signals involved in skeletal muscle regeneration. Cell Rep 2020;30:3583, e5–3595. 32160558 10.1016/j.celrep.2020.02.067 PMC7091476

[38] Alcalde‐Estévez E , Asenjo‐Bueno A , Sosa P , Olmos G , Plaza P , Caballero‐Mora MÁ , et al. Endothelin‐1 induces cellular senescence and fibrosis in cultured myoblasts. A potential mechanism of aging‐related sarcopenia. Aging 2020;12:11200–11223. 32572011 10.18632/aging.103450 PMC7343454

[39] Wolf U , Eckert S , Walter G , Wienke A , Bartel S , Plontke SK , et al. Prevalence of oropharyngeal dysphagia in geriatric patients and real‐life associations with diseases and drugs. Scientific Rep 2021;11:1–14. 10.1038/s41598-021-99858-w PMC8578645 34754078

[40] von Haehling S , Morley JE , Coats AJS , Anker SD . Ethical guidelines for publishing in the Journal of Cachexia, Sarcopenia and Muscle: update 2021. J Cachexia Sarcopenia Muscle 2021;12:2259–2261. 34904399 10.1002/jcsm.12899 PMC8718061

