# Supplementary material for: A negative feedback loop between fibroadipogenic progenitors and muscle fibres involving endothelin promotes human muscle fibrosis
Source: J Cachexia Sarcopenia Muscle. 2022 Mar 22;13(3):1771–84. doi: 10.1002/jcsm.12974 (PMC9178170; doi:10.1002/jcsm.12974)
Supplement: Supplementary file 4 — Fig S1 Expression of FAP markers by CyTOF. Mass cytometry contour plots coloured by density showing the gating strategy for the PDGFRα, CD90 and CD105 markers for each individual from which MCT, FibMCT and FibMOP muscle biopsies were obtained. Fig S2 CD106, PDGFRβ and LAP (TGFβ1) expression in FAP cells from fibrotic and nonfibrotic muscles by CyTOF. UMAP plots showing the expression patterns of (a) CD106 (VCAM1), (b) PDGFRβ and (c) LAP (TGFβ1) in nonmyogenic cells from MCT FibMCT and FibMOP muscle biopsies. The cells are coloured according to the intensity with which the marker was expressed. For CD106 and PDGFRβ the gating strategy for each patient is highlighted. A histogram recapitulating those percentages under the different conditions is also shown (***P < 0.001, ****P < 0.0001). Fig S3 Comparison of CD56‐ cells from fibrotic and nonfibrotic muscles. (a) Live imaging video of CD56‐ cells from MCT, FibMCT and FibMOP muscle biopsies in growth medium with 20% serum. (b) Representative nuclear staining of basal pSMAD3 + expression on serum‐starved FAPs from MCT, FibMCT and FibMOP muscle biopsies (n = 3 biological replicates). (c) Additional view of the PCA presented in Figure 3 highlighting specifically PC1 versus PC2 with all cell types (left) and PC2 versus PC3 with FibMCT and FibMOP only (right). MCT (yellow), FibMCT (orange) and FibMOP (red) muscle biopsies. Each dot represents cells from one patient. Fig S4 ECM secretion from nonfibrotic and fibrotic muscles. (a) RT‐qPCR quantification of FBN2 and MATN2 gene expression normalized to B2M expression in FAPs from MCT, FibMCT and FibMOP muscle biopsies (n = 3 biological replicates). (b) Immunofluorescence analysis of Dystrophin (green), Hoechst (blue) and Collagen7a1 (red) was performed on non‐fibrotic (MCT) and fibrotic (FibMOP) muscles. (c) Experimental scheme used to inject FAPs isolated from MCT, FibMCT and FibMOP muscle biopsies into the regenerating TA muscle of immunodeficient mice. A total of 1.4 × 10e ce [file JCSM-13-1771-s003.pdf]

FigS1

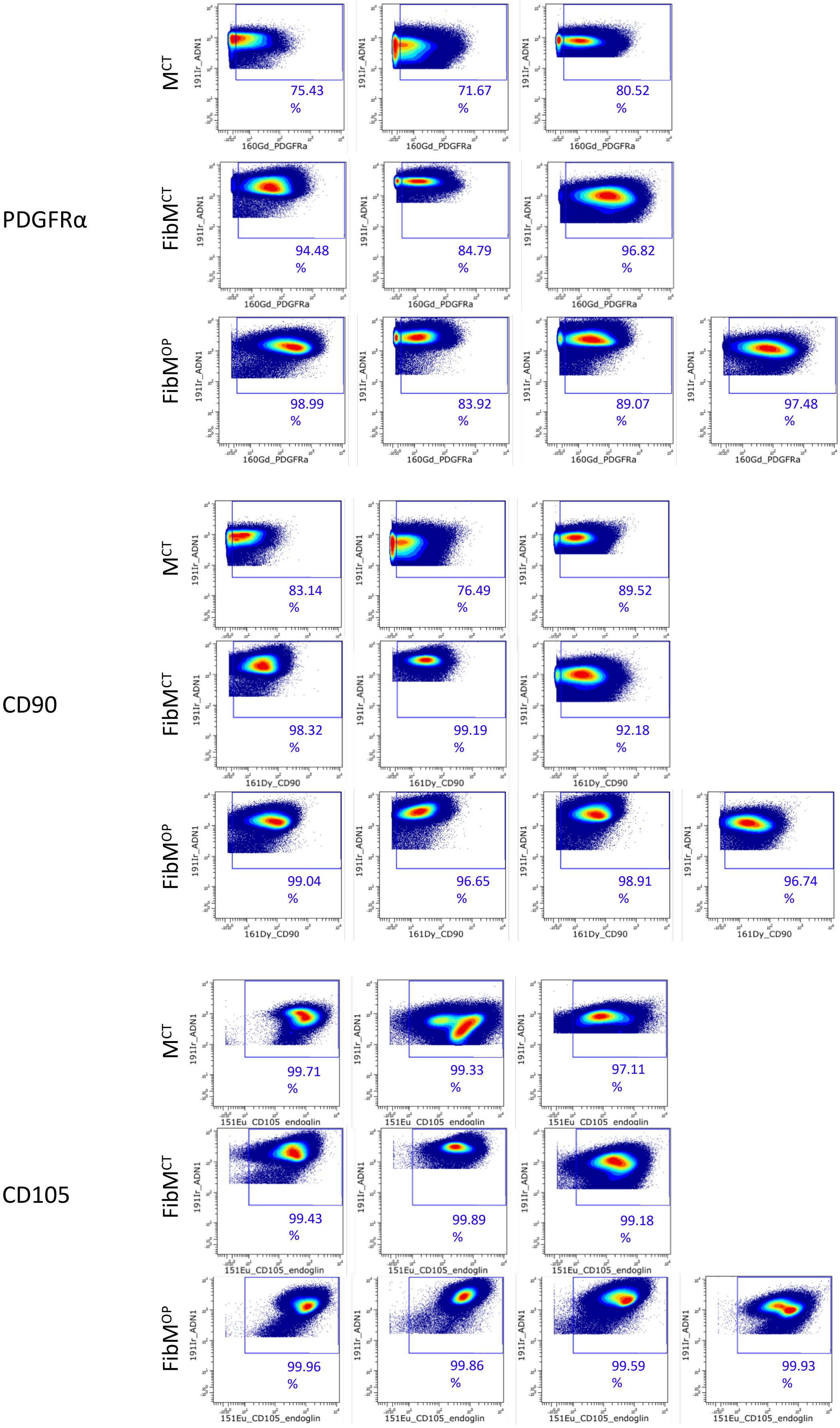

PDGFRα
MCT
75.43 %
71.67 %
80.52 %
FibMCT
94.48 %
84.79 %
96.82 %
FibMOP
98.99 %
83.92 %
89.07 %
97.48 %
160Gd_PDGFRa
191Ir_ADN1
CD90
MCT
83.14 %
76.49 %
89.52 %
FibMCT
98.32 %
99.19 %
92.18 %
FibMOP
99.04 %
96.65 %
98.91 %
96.74 %
161Dy_CD90
CD105
MCT
99.71 %
99.33 %
97.11 %
FibMCT
99.43 %
99.89 %
99.18 %
FibMOP
99.96 %
99.86 %
99.59 %
99.93 %
151Eu_CD105_endoglin

FigS2

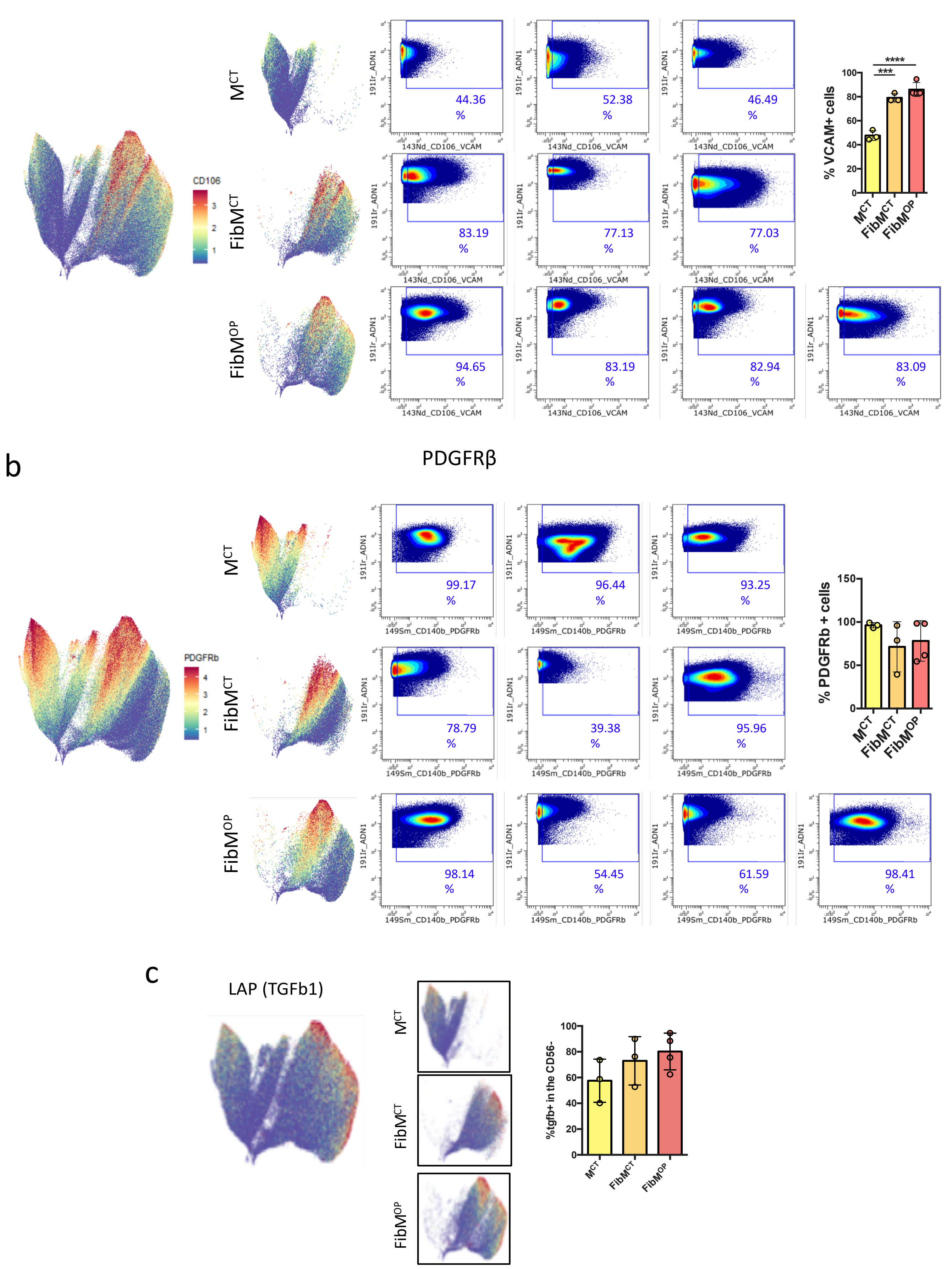
a
VCAM1 (CD106)
CD106
MCT
FibMCT
FibMOP
191Ir_ADN1
143Nd_CD106_VCAM
44.36 %
52.38 %
46.49 %
83.19 %
77.13 %
77.03 %
94.65 %
83.19 %
82.94 %
83.09 %
% VCAM+ cells
****
***
b
PDGFRβ
PDGFRb
149Sm_CD140b_PDGFRb
99.17 %
96.44 %
93.25 %
78.79 %
39.38 %
95.96 %
98.14 %
54.45 %
61.59 %
98.41 %
% PDGFRb + cells
c
LAP (TGFb1)
%tgfb+ in the CD56-

FigS3

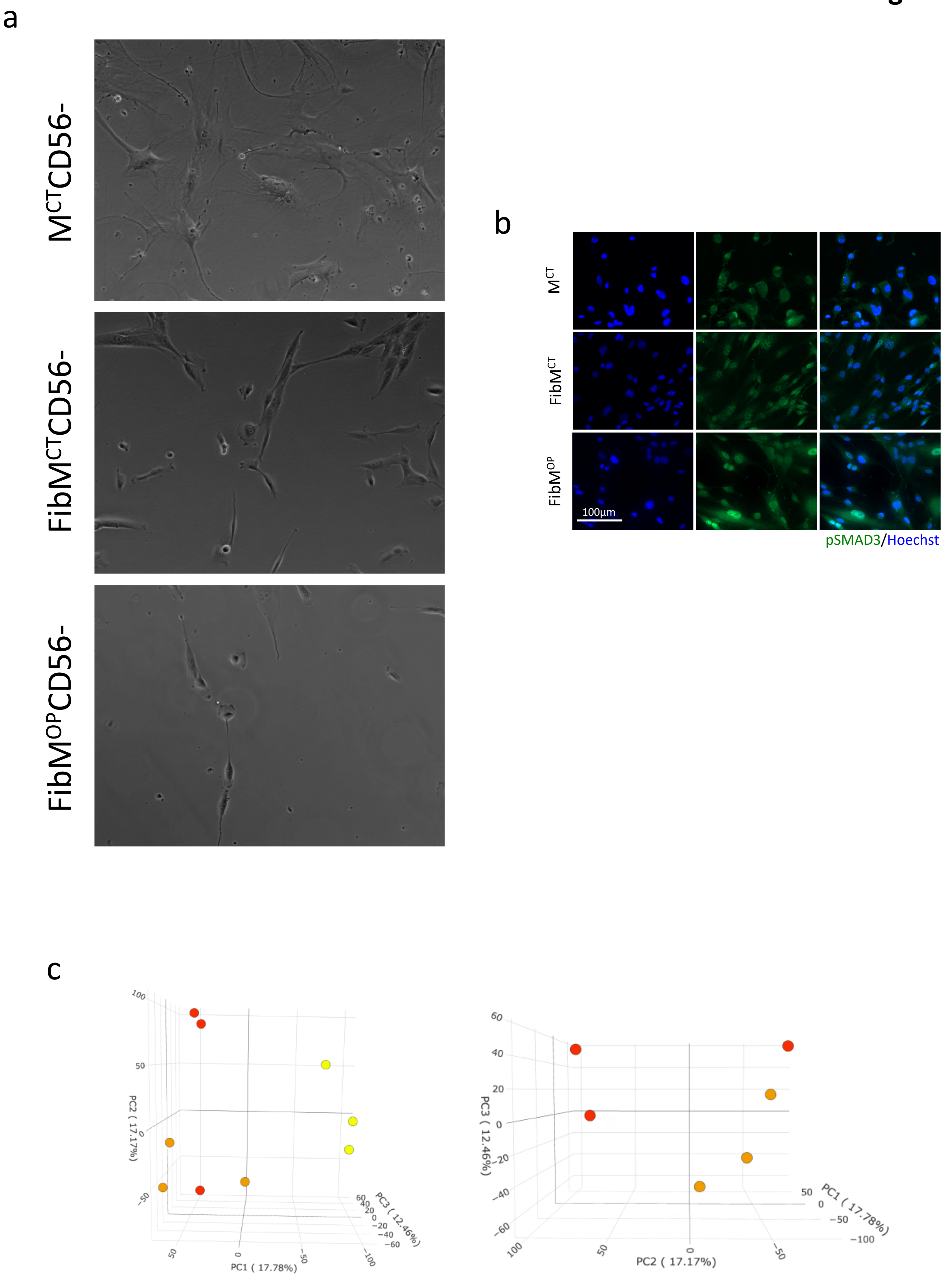

a
MCTCD56-
FibMCTCD56-
FibMOPCD56-
b
MCT
FibMCT
FibMOP
100µm
pSMAD3/Hoechst
c
PC1 (17.78%)
PC2 (17.17%)
PC3 (12.46%)
PC2 (17.17%)
PC3 (12.46%)
PC1 (17.78%)

FigS4

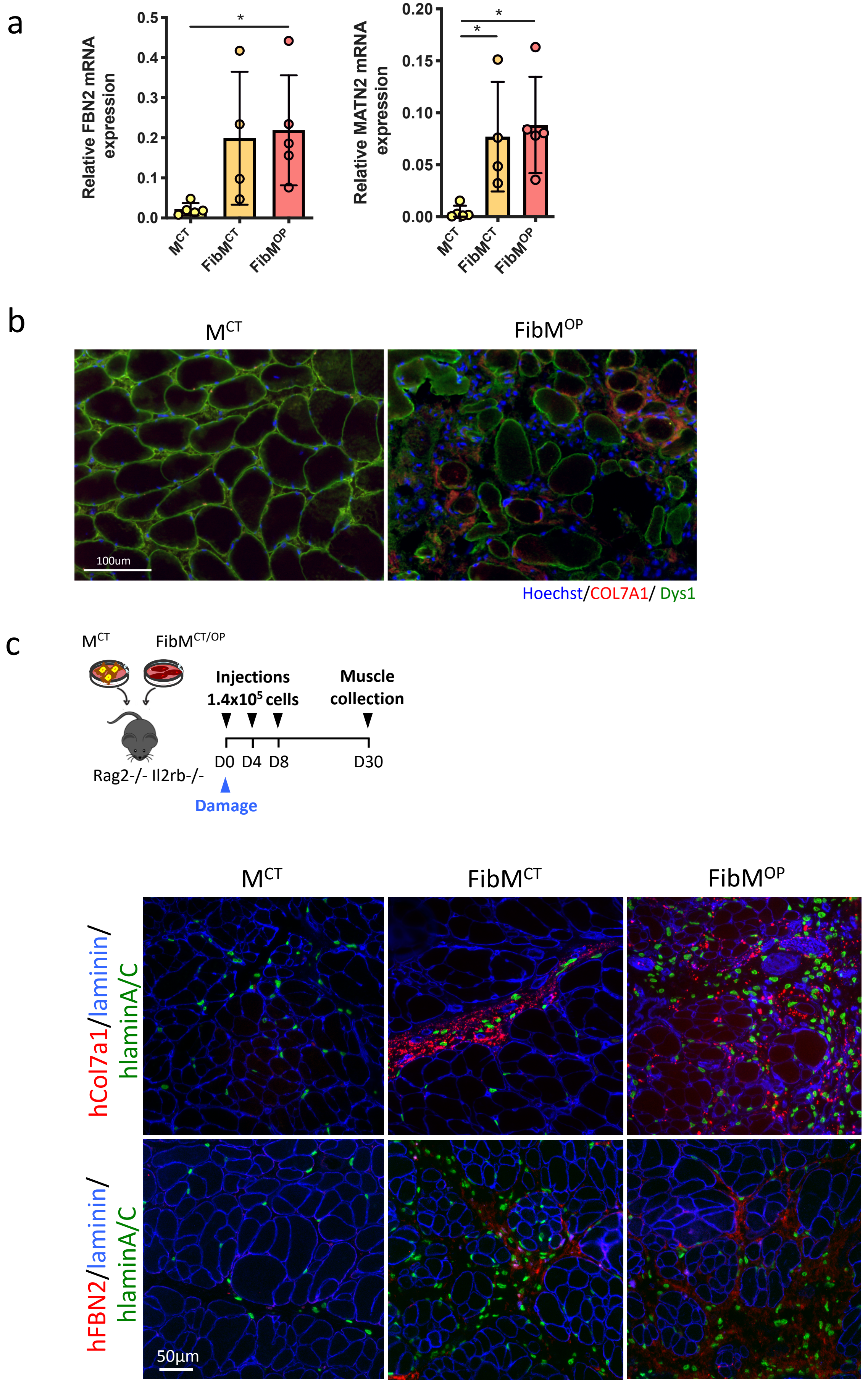
a
Relative FBN2 mRNA expression
0.5
0.4
0.3
0.2
0.1
0.0
*
M^CT
FibM^CT
FibM^OP
Relative MATN2 mRNA expression
0.20
0.15
0.10
0.05
0.00
b
M^CT
FibM^OP
100um
Hoechst/COL7A1/ Dys1
c
M^CT
FibM^CT/OP
Rag2-/- Il2rb-/-
Injections
1.4x10^5 cells
Muscle
collection
D0
D4
D8
D30
Damage
M^CT
FibM^CT
FibM^OP
hCol7a1/laminin/hlaminA/C
hFBN2/laminin/hlaminA/C
50µm

FigS5

a

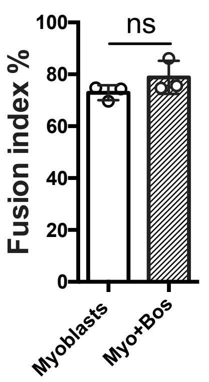
ns
Fusion index %
100
80
60
40
20
0
Myoblasts
Myo+Bos

b

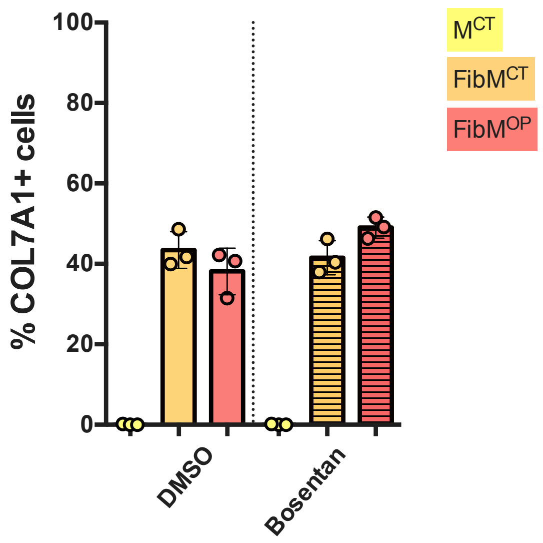
% COL7A1+ cells
100
80
60
40
20
0
DMSO
Bosentan
MCT
FibMCT
FibMOP

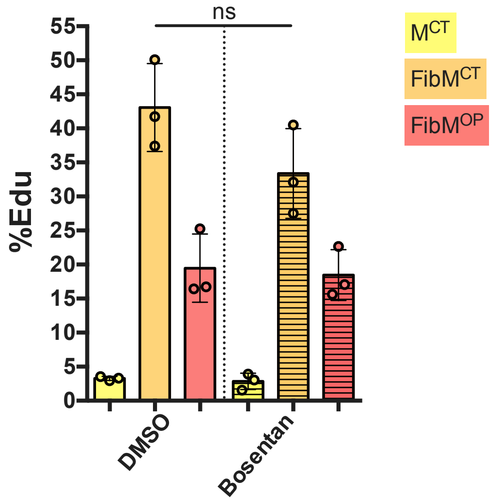
ns
%Edu
55
50
45
40
35
30
25
20
15
10
5
0
DMSO
Bosentan
MCT
FibMCT
FibMOP
